# The viewpoint by White and colleagues critiquing the evaluation of the safety and efficacy of mass chemotherapy for *Taenia solium* taeniasis is unsubstantiated

**DOI:** 10.1371/journal.pntd.0008592

**Published:** 2020-09-03

**Authors:** Michelle M. Haby, Leopoldo A. Sosa Leon, Ana Luciañez, Ruben Santiago Nicholls, Ludovic Reveiz, Meritxell Donadeu

**Affiliations:** 1 Department of Chemical and Biological Sciences, University of Sonora, Hermosillo, Sonora, Mexico; 2 Centre for Health Policy, Melbourne School of Population and Global Health, The University of Melbourne, Melbourne, Victoria, Australia; 3 Independent Consultant, Hermosillo, Sonora, Mexico; 4 Neglected Infectious Diseases, Communicable Diseases and Environmental Determinants of Health, Pan American Health Organization/World Health Organization, Washington DC, United States of America; 5 Department of Evidence and Intelligence for Action in Health, Pan American Health Organization/World Health Organization, Washington DC, United States of America; 6 Faculty of Veterinary and Agricultural Sciences, The University of Melbourne, Melbourne, Victoria, Australia; 7 Initiative for Neglected Animal Diseases (INAND), Midrand, South Africa; George Washington University School of Medicine and Health Sciences, UNITED STATES

White et al. have raised a number of concerns about the findings of our systematic review [1]. However, we do not believe they have substantiated their claims with quality research evidence. Our systematic review aimed to assess the best available scientific evidence concerning the effectiveness and safety of different drugs in preventive chemotherapy for *Taenia solium* taeniasis in endemic populations. The advantage of systematic reviews over selective reporting of the research evidence is that they seek to minimize bias and use transparent methods to find all research that meets pre-defined inclusion criteria. Our systematic review used the highest quality systematic review methods [2] and the limitations of the review and of the included studies are discussed. We note in the abstract, results and discussion that the results have a low certainty of evidence due to a high risk of bias in the included studies and heterogeneity in combined estimates. We also discuss the issues regarding the lack of sensitivity of microscopy and non-use of a species-specific diagnostic test (we present the different diagnostic methods used in S1 Table of the paper) [3]. We believe that we have presented a transparent and balanced account of the available evidence.

In relation to some of the specific points raised by White et al. we note that we have presented the meta-analysis results stratified by drug and dose to allow the reader to identify any important differences in effectiveness, which was the aim of the review. It was not possible to further stratify the results by the diagnostic test used due to the relatively small number of studies. However, more important than the diagnostic tests used is the fact that all studies had a high risk of bias due to issues such as lack of randomization, incomplete outcome data and lack of blinding of outcome assessors [3]; a fact also acknowledged in the abstract by the statement that the “findings have a low certainty of evidence.” And this low certainty of evidence is independent of drug and dose used.

The possibility that albendazole (ALB) does not kill the *T*. *solium* scolex and egg shedding may still occur after treatment is raised by White et al. 2020, though not substantiated with research evidence. We were unable to find evidence to confirm this statement but note the findings of one study of mass drug administration (MDA) with ALB 400mg triple dose included in the systematic review that used both Copro-PCR and Co-Ag-ELISA [4]. The taeniasis results could not be included in the meta-analysis because interventions in pigs were also applied, thus making it difficult to attribute the results to the MDA. However, this study notes that: “None of the 11 taeniasis carriers identified in [pre-October] 2013 remained positive in [January] 2015.” This suggests that the MDA with ALB did kill the scolex as the January 2015 measurement was taken 10 months after the second MDA, which gives more than sufficient time for the scolex to re-grow a mature tapeworm and produce eggs had the scolex remained.

In relation to the analysis of side-effects, White et al. 2020 quote two publications. One is a case report of neurological side-effects after MDA with 5mg/kg praziquantel [5]. The other contains merely speculation about what might be the cause of seizures associated with onchocerciasis [6] and neither were studies of MDA for schistosomiasis as incorrectly stated by White et al. 2020. We excluded case reports from our systematic review because they have a very low level of evidence of effect, and chance cannot be ruled out [7, 8]. Instead, 11 studies met the inclusion criteria and were included in the systematic review (Table 4 in [3]), with a combined participation of over 17,000 individuals. In addition, we conducted a supplementary search for studies reporting side-effects from treatment with any of the three drugs (results presented in S4 File) and the results are reported in the discussion [3].

White et al. also note that neurological side-effects would be anticipated to occur 3 to 5 days after treatment but, again, they did not cite any evidence to support this claim and we were unable to find any supporting evidence for their statement. In fact, those studies that do note the time of occurrence of side-effects generally report effects within the first 1–3 days [9, 10] following treatment, and in the case report cited by White et al. symptoms started within 24 hours of treatment [5].

We encourage constructive criticism of all research, including our own, but we suggest that this should be based on research evidence rather than reliance on perception and opinion. *T*. *solium* endemic countries need help to control the problem. To make decisions about possible solutions, they need an impartial summary of the best available evidence of effectiveness and safety, along with an assessment of other factors important for decision-making, including whether the problem is a priority, a rational assessment of the balance between desirable and undesirable effects, values and preferences, resource requirements, impact on equity, feasibility, and acceptability [11, 12].

## Disclaimer

Some authors are staff members of the Pan American Health Organization. The authors alone are responsible for the views expressed in this publication, and they do not necessarily represent the decisions or policies of the Pan American Health Organization.
